# Inferring fish behaviour at the trawl mouth from escape location

**DOI:** 10.7717/peerj.14746

**Published:** 2023-01-25

**Authors:** Vang Y. Nguyen, Shannon M. Bayse, Haraldur Arnar Einarsson, Ólafur Arnar Ingólfsson

**Affiliations:** 1 Fisheries and Marine Institute, Memorial University of Newfoundland, St. John’s, Newfoundland and Labrador, Canada; 2 Marine and Freshwater Research Institute, Hafnarfjörður, Iceland; 3 Institute of Marine Research, Nordnes, Bergen, Norway

**Keywords:** Fish behaviour, Herding behaviour, Response behaviour, Swimming capacity, Roundfish, Flatfish, Monkfish, Catch comparison, Collection bags, Rockhopper gear

## Abstract

In this study, we used escape location underneath the trawl to understand groundfish herding behaviour at the trawl mouth. Three collecting bags (port, center, starboard) were mounted under the trawl and behind the footgear to collect escapees. The escape-at-length of species that escaped into the center bag were compared to the two wing bags to infer fish response behaviour, herding behaviour, and swimming capacity at the trawl mouth. For roundfish, smaller-sized individuals escaped more in the center for both Atlantic cod (*Gadus morhua*) and haddock (*Melanogrammus aeglefinus*), <20 and 11 cm, respectively, indicating that larger-sized fish were to a greater extent seeking to escape under the trawl at the wings, *vs* small fish being herded to the center and likely overrun due to reduced swimming capacity. For flatfish and monkfish (*Lophius piscatorius*), results varied. European plaice (*Pleuronectes platessa*), American plaice (*Hippoglossoides platessoides*), and monkfish were caught most often in the wings, though not significantly for American plaice. Catches of dab (*Limanda limanda*) between 18 and 27 cm were significantly higher in the center, with no difference for smaller and larger individuals. The differences between fish escape location likely result from a combination of varying herding behaviour, size, and swimming capacity. Here, we were able to show how these size-dependent behaviours relate to fish response behaviour, escape behaviour, size, and likely swimming capacity.

## Introduction

Fish reactions to trawl components could alter fish’s herding and escape patterns, directly affecting catch efficiency. Several investigations have revealed that most fish in front of the trawl are herded into the trawl path by visual cues and trawl components (doors, bridles, and footgear), thus becoming available for capture (Ryer, 2008; Wardle, 1993; Winger, Eayrs & Glass, 2010). Fish behaviour during the herding process, particularly at the mouth of the trawl, is a critical process when considering how to improve and understand trawl selectivity (Engås & Godø, 1989; Godø & Walsh, 1992). During the herding process, fish react to the advancing trawl components in a way that is dependent on their swimming capacity and endurance, visual range, and physiological conditions, which can vary among species and differ according to size (Beamish, 1966; He, 1991; Winger, He & Walsh, 1999). This leads to different behavioural patterns of fish at the trawl mouth, which in turn results in fish either falling back into the trawl net or escaping under the fishing line or over the headline of the trawl (Winger, Eayrs & Glass, 2010).

Roundfish reactions have been observed to approaching trawl components (Pitcher & Parrish, 1993; Wardle, 1993). These reactions include moving closer to the seabed and swimming away from trawl doors and bridles to keep the approaching threats within visual range, known as the “fountain maneuver” pattern (Wardle, 1993), which herds fish into the trawl path (Winger, Eayrs & Glass, 2010). Once fish reach the trawl mouth (*i.e*., trawl opening), they alter their course and swim in the opposite direction of the tow, in front of the trawl (Winger, Eayrs & Glass, 2010).

Several studies have shown that roundfish behaviour in the trawl mouth varies among species. For example, Atlantic cod (*Gadus morhua*) maintain a position close to the seabed, where haddock (*Melanogrammus aeglefinus*) tend to rise in the water column (Godø & Walsh, 1992; Main & Sangster, 1981). Furthermore, the response of roundfish, such as Atlantic cod and haddock, at the trawl mouth was size-dependent (Godø, 1994). Valdemarsen, Engås & Isaksen (1985) found that small cod and haddock entered the trawl at heights closer to the seabed than larger individuals. A follow-up study by Engås & Godø (1989) revealed length-dependent escape, where more small cod and haddock escaped underneath a survey trawl’s fishing line than larger fish. Investigations supported this length-dependent behaviour at the trawl mouth, where larger fish with greater swimming endurance can keep their station for long periods and find escape routes compared to small fish which tend to seek escape under the fishing line (Ingólfsson & Jørgensen, 2006; Ryer, 2008; Wardle, 1993).

Behavioural observations of flatfishes show that individuals respond to the advancing trawl at a shorter distance than roundfish, and reactions are characterized by an anti-predator strategy (Main & Sangster, 1981; Ryer, 2008). The initial reaction is to conceal themselves with camouflage, which combines burying themselves in sediment, cryptic coloration, and reducing activity to minimize their detection by a predator (Gibson, 2005). When the bridles of the trawl advance closer, fish either remain immobile, allowing the bridles to pass above them, or swim at a 90° angle into the trawl path (Bryan et al., 2014; Main & Sangster, 1981; Ryer, 2008; Winger, Eayrs & Glass, 2010). Some fish that swim slower than the speed of the advancing bridle are available to escape underneath, while others that swim equal to or greater than the coming bridle, can reach the trawl path and become available for capture (Main & Sangster, 1981; Ryer & Barnett, 2006; Ryer, Rose & Iseri, 2010). At the trawl mouth, reactions in front of the footgear are typically brief, often less than 1 min (Ryer, 2008), but can be greater than 5 min (Bayse, Pol & He, 2016), and then fish either flip and fall back into the net at the heights less than 1 m from the seabed or escape under the footgear (Bublitz, 1996; Bayse, Pol & He, 2016).

Different escape patterns underneath the trawl, between and within species, have been quantified using different methods. Starting in the 1980’s, underwater camera technology was used to investigate fish behaviour at the trawl mouth (Main & Sangster, 1981). Qualitative descriptions of fish behaviour continued through the 1990’s (Godø, 1999; Walsh & Hickey, 1993). Increasingly, quantitative techniques have been used to describe fish behaviour at the trawl mouth that lead fish either escaping under or entering the trawl (Albert, Harbitz & Høines, 2003; Bayse, Pol & He, 2016; Underwood et al., 2015). Additionally, the use of underwater video in combination with the catch data has shown potential for quantifying fish behaviour while herding (Godø, 1999; Weinberg & Munro, 1999; Larsen et al., 2018).

The bottom trawl includes a weighted footgear at the bottom of the trawl mouth to keep the trawl in contact with the seabed and protect the netting from damage (Montgomerie, 2022). The form of footgear is dependent on the type of bottom trawl, seabed, and targeted species. Recently, the rockhopper footgear has been widely used in commercial trawl fisheries to allow fishing on rougher bottoms, reduce net damage, and can improve capture efficiency (Engås & Godø, 1989; Ingólfsson & Jørgensen, 2006). Additionally, rockhopper footgear has been shown to be more effective in catching fish close to the bottom relative to traditional steel bobbins gear (Engås & Godø, 1989; Main & Sangster, 1985). By using bigger rubber spacers between the rockhopper discs, the inter-disc spaces can be increased to facilitate escape of small fish under the footgear (Engås & Godø, 1989; Walsh, 1992). For instance, the escape rates of gadoid species have been observed to be length-dependent (Engås & Godø, 1989; Ingólfsson & Jørgensen, 2006).

The purpose of this study was to infer fish response behaviour, herding behaviour, and swimming capacity at the trawl mouth by comparing retained fish between collecting bags under the trawl. Fish escape was considered a consequence of fish behaviour or response to the herding effect of the trawl (Walsh, 1992). Additionally, the effectiveness of fish behaviour (*i.e*., ability to escape) at the trawl mouth may differ according to fish size, resulting in differences in length-based escape under the footgear at particular locations. However, most works on fish behaviour at the trawl mouth have focused on the center area, and less quantitative work has been focused on the wings. Here, we used escape-at-length comparison data to describe and quantify the extent to which fish behaviour sets limits to fish escape ability along the footgear. This study provides additional insights into the length-dependent behaviour of fish at different areas of the trawl mouth during the herding process, which are currently unclear, and important for further development and understanding of bottom trawl selectivity and fish behaviour.

## Materials and Methods

### Sea trials

**Figure 1:**
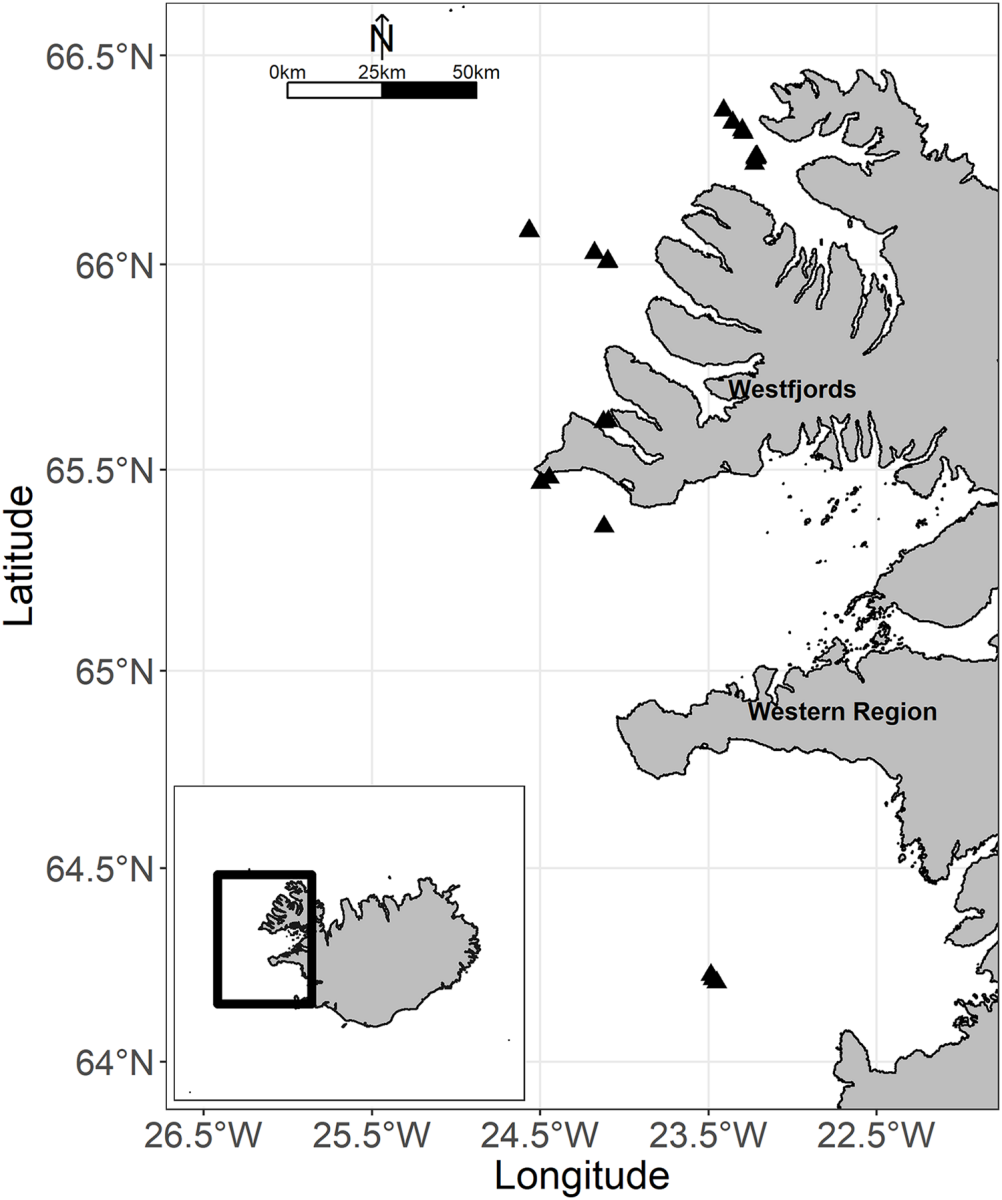
Map of the study area off western Iceland. The black triangles showed all locations of tows. Map credit: global administrative areas (https://gadm.org/); license: GADM license.

Sea trials were conducted onboard the research vessel “Árni Friðriksson” (70 m, 4 × 1,000 kW) from 7 to 13 September 2009. The fishing grounds were off the Westfjords and in Faxaflói Bay Iceland (Fig. 1). Fishing was carried out during day and night (between 22:20 (sunset) and 4:30 (sunrise), based on the local time zone of the study area during sea trials (see timeanddate.com)). Tow duration was defined from when the gear was on the bottom (estimated by echograph) till the start of haulback. Fishing locations were chosen in collaboration with the captain such that rough bottoms were avoided due to the vulnerability of the collecting bags to damage. Gear performance (towing velocity, duration, warp length, and door spread) was recorded for each tow.

### Gear specifications

**Figure 2:**
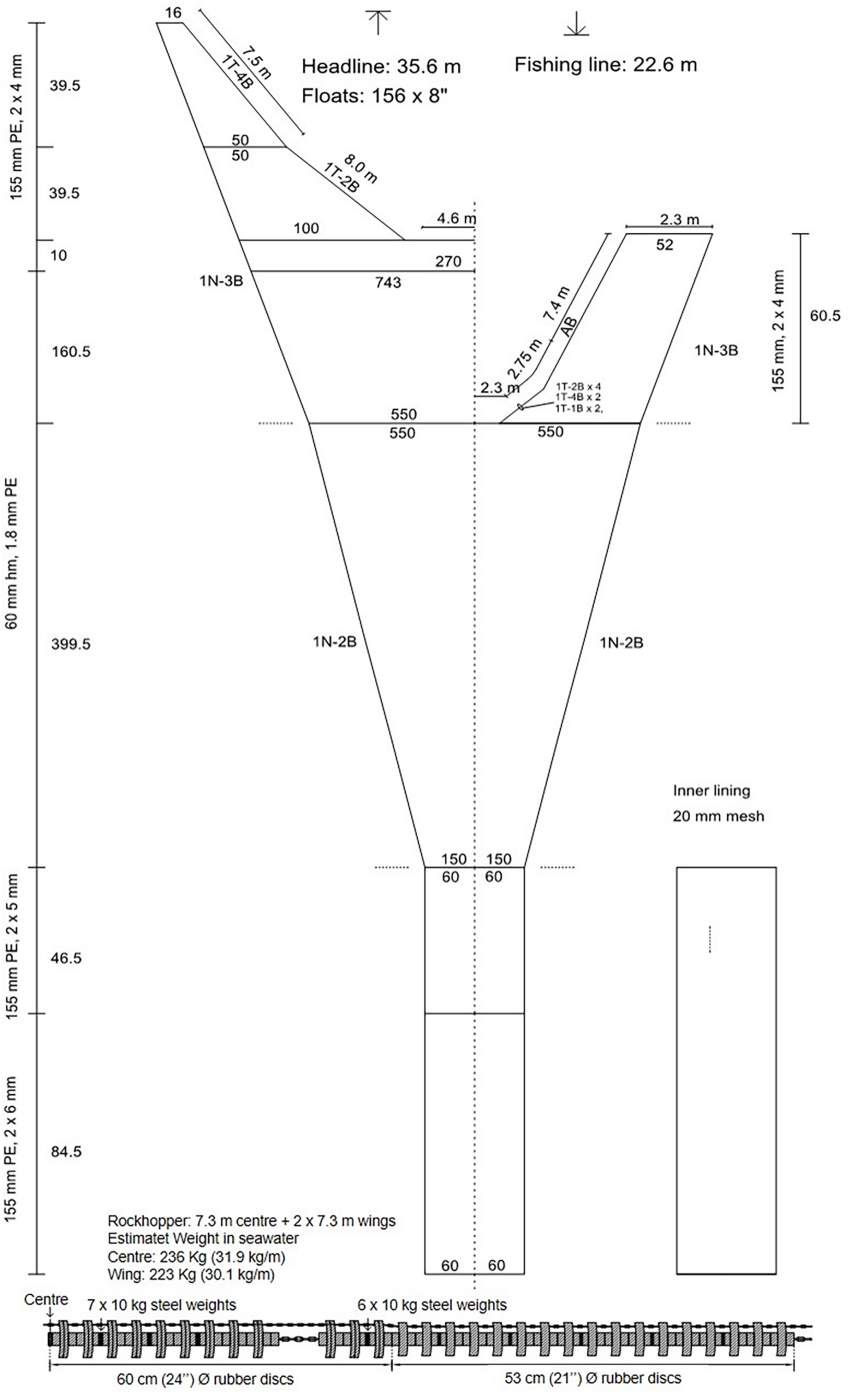
Net plan of the Gulltoppur trawl.

**Figure 3:**
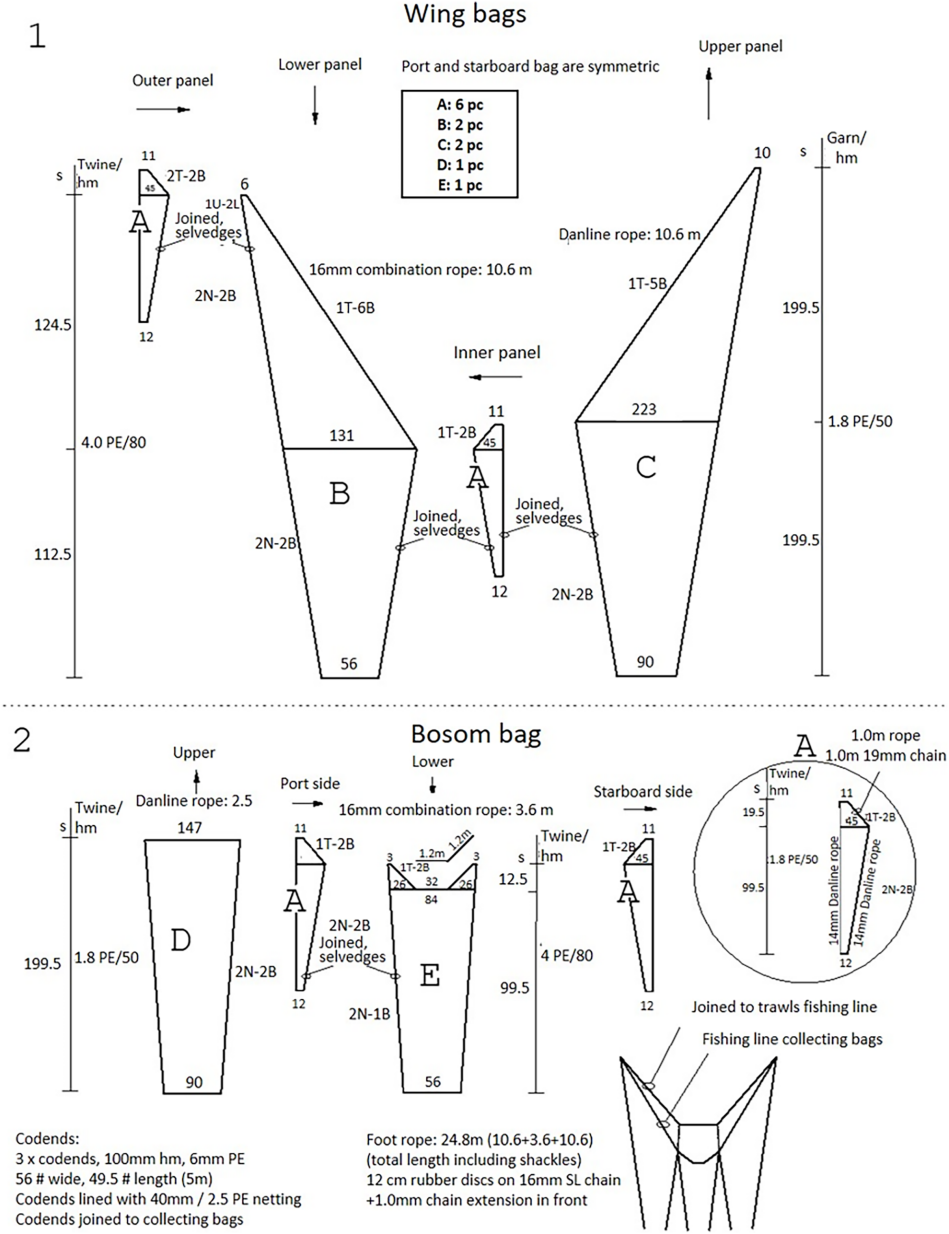
The net plan of the collecting bags used to capture fish that escaped under the trawl. (1) Illustrates the wing bags, and (2) illustrates the bosom bag.

The trawl used was the “Gulltoppur”, a design used by many Icelandic stern trawlers (Fig. 2). The trawl doors were of the type Poly-Ice no. 8, 2,700 kg. Backstraps were 9 m long, sweeps 65 m, bridles 67 m and ground gear extensions 13 m. The total distance from doors to the ground gear was 154 m. A commercial rockhopper footgear was used. It had 60 cm diameter discs at the center and the three rearmost discs of the wing. The rest of the wing had 53 cm discs. The gear weight in seawater was approximately 30 kg m^−1^. Small mesh (20 mm) collecting bags were used to retain all the fish that escaped underneath the fishing line of the trawl (Ingólfsson & Jørgensen, 2006). Three collecting bags were attached to the footgear, including a center bag, and two wing bags (Fig. 3). For each collecting bag, the headline of the collecting bag was joined to the fishing line of the trawl. The fishing line of the collecting bags had a footgear made of 12 cm rubber discs threaded on a 24.8 m long 16 mm steel chain. A 1.0 m long chain extension, made of 19 mm steel, was used to connect the front ends of the gear to the front ends of the fishing line (Fig. S1). There were no gaps between the bags.

### Catch sampling

Catches from the collecting bags and codend were processed separately. Individuals were counted and measured to the nearest centimeter below total length. Subsampling was applied if a large number of a species was encountered. Tows that had less than 10 observations in collecting bags were removed from the analysis for the specific species.

### Data analysis

This study compared the escape-at-length of each species that escaped between the center and wing sections of the footgear. This escape-at-length comparison is performed using the *Center/*(*Center + Wing*) function, where *Center* is the number of fish measured in the center bag, and *Wing* is the number of fish measured in both wing bags, per length class (cm). The function estimates the proportion at length in catch from the center bag as a proportion of the total count at length from center and wing bags. As proportional data, it is considered binomial.

The escape-at-length data was modeled following procedures similar to Holst & Revill (2009) and Eighani et al. (2020). The curves of the proportions (logit; (*Center/Center + Wing*)) were modeled with low-order orthogonal polynomials (0 to 4th degree) using Generalized Linear Mixed Models (GLMMs) in R statistical software (R Development Core Team, 2009). The dependent variable was the logit (*Center/*(*Center + Wing*); catch proportion at length), the independent variable was fish length (*MLL*), and the subsample ratio between *Center* and *Wing* was considered as an offset. The random effect of *Tow* was added on either or both intercept and slope of the models to account the variations in the escape-at-length data among tows due to the effects of extrinsic factors (*i.e*., environmental conditions, fish density, *etc*.) and/or intrinsic factors (*i.e*., differences in individual fitness of same length class between tows). The restricted maximum likelihood (REML) method was used to fit the models using the glmer function of the lme4 package (Bates et al., 2015). The model equation was therefore:

${\rm Logit} \, (Center/(Center+Wing)) = {{\rm log}\, {(q_b/q_w)} + {\beta_0} + {\beta_1 MLL} + {\ldots} + {\beta_k MLL^k} + {\in}}$

where *q*_*b*_ and *q*_*w*_ are the subsampling ratios for *Center* and *Wing*, $\beta_0$ is the intercept. *$\beta$* values are the model parameters, increasing from 1 to k = 4, corresponding to increasing the polynomial order from linear polynomial to 4^th^ degree polynomial. $\epsilon$ is the between haul random variable, where $\epsilon$ ~ N (0, σ^2^). For each model above a 1^st^ degree polynomial, the independent variables were rescaled to prevent correlation from polynomial terms as orthogonal polynomials using the *poly* function in the stats package. A random effect was used originally on each polynomial term. However, if convergence problems or singularity were observed, the random effect was removed from the linear term to enable proper model fit. DHARMa was used to investigate model dispersion and residual diagnostics (Hartig, 2021).

The proportion values were estimated to be between 0 and 1 per length class. For example, a proportion of 0.5 means that the same retention rate was observed between the center and wings for a particular length class. Additionally, if a proportion of 0.75 was observed, it means 75% of individuals escaped in the center and 25% in the wings at a specific length. Model selection was based on the minimum Akaike information criterion value (Akaike, 1974) with a correction for small sample sizes (AICc). It was calculated using the AICctab function in the bbmle package (Bolker, 2021). The best model was chosen with the lowest AICc value. If there were multiple models within 2 AICc, the simplest model was considered as the best model. The best model selected for each species was then used to investigate the effects tows at day or night (diel effect) on the escape-at-length of species between locations if observed in at least five tows in each treatment (*i.e*., day and night tows). The diel effect was added in the model as the independent variable. If the diel effect significantly affected size selectivity was determined by a likelihood ratio test, where the test statistic ( $\chi^{2}$) determined the difference in deviance between the best model and the best model containing the diel effect at an α of 0.05.

The final model’s confidence intervals (CIs) were estimated using the bootMer mixed model bootstrapping function in lme4 and the boot.ci function in the boot package (Canty & Ripley, 2021) with 1,000 simulations. The CIs were used to determine the significant difference in proportion at length retained between the center and wings. If the CIs included 0.5 at a particular length, there is no significant difference between escape locations.

This study was performed by the Marine and Freshwater Research Institute, which complies with the regulation imposed by the Icelandic “Food ministry”. This regulation allows the landing of all fish catch during sea trials.

## Results

A total of 34 tows were made. Nine tows were removed due to sampling inconsistencies. Thus, 25 valid tows were used in the subsequent analyses, including 20 tows during the day and five at night. Tow speeds were 3.6 kn on average (range: 3.5–3.9 kn), and the average tow duration was 44 min (range: 23–79 min). The warp length was 207.0 m (range: 122.5–484.6 m), and the door spread was 58.5 m (range: 43 to 96 m). Tows were conducted at depths ranging from 26 to 192 m, with a median depth of 73 m.

A total of 31 species were observed. However, six were found in sufficient abundance to be used in the analyses, including roundfish (Atlantic cod and haddock), flatfish (European plaice (*Pleuronectes platessa*), American plaice (*Hippoglossoides platessoides*) and dab (*Limanda limanda*)) and monkfish (*Lophius piscatorius*). Atlantic cod was the most frequently occurring species and observed in 25 valid tows, including 19 day tows, five night tows, and one tow was carried out in both day and night. The second most frequent species were haddock and European plaice, observed in 23 (17 day tows, five night tows, and one tow was carried out in both day and night) and 22 (18 day tows and four night tows) valid tows, respectively, followed by American plaice, observed in 10 valid tows (Seven day tows and three night tows). Dab and monkfish observed in nine valid tows (six day tows and three night tows) (Table 1). One tow was subsampled on the port wing bag for Atlantic cod, haddock, and American plaice. Subsampling occurred on one tow, on the center bag for European plaice. For subsampling tows, at least 55% fish or more were measured, except for American plaice, whose subsampling was 25%. No tows were subsampled for dab and monkfish. For species that had at least five tows for day and night, no model showed a significant effect (*p* > 0.05) for fishing at night or day.

**Table 1:** Overview of valid tows observed for each species.

Measurement	Cod	Haddock	European plaice	American plaice	Dab	Monkfish
No. of fish in bosom bag	1,689	1,585	739	279	220	61
No. of fish in wing bags	2,105	1,590	1,219	406	185	216
Total no. of fish measured	3,794	3,175	1,958	685	405	277
No. of tow	25	23	22	10	9	9
Min. length (cm)	6	6	10	7	6	23
Max. length (cm)	109	50	65	46	41	83

### Atlantic cod

**Figure 4:**
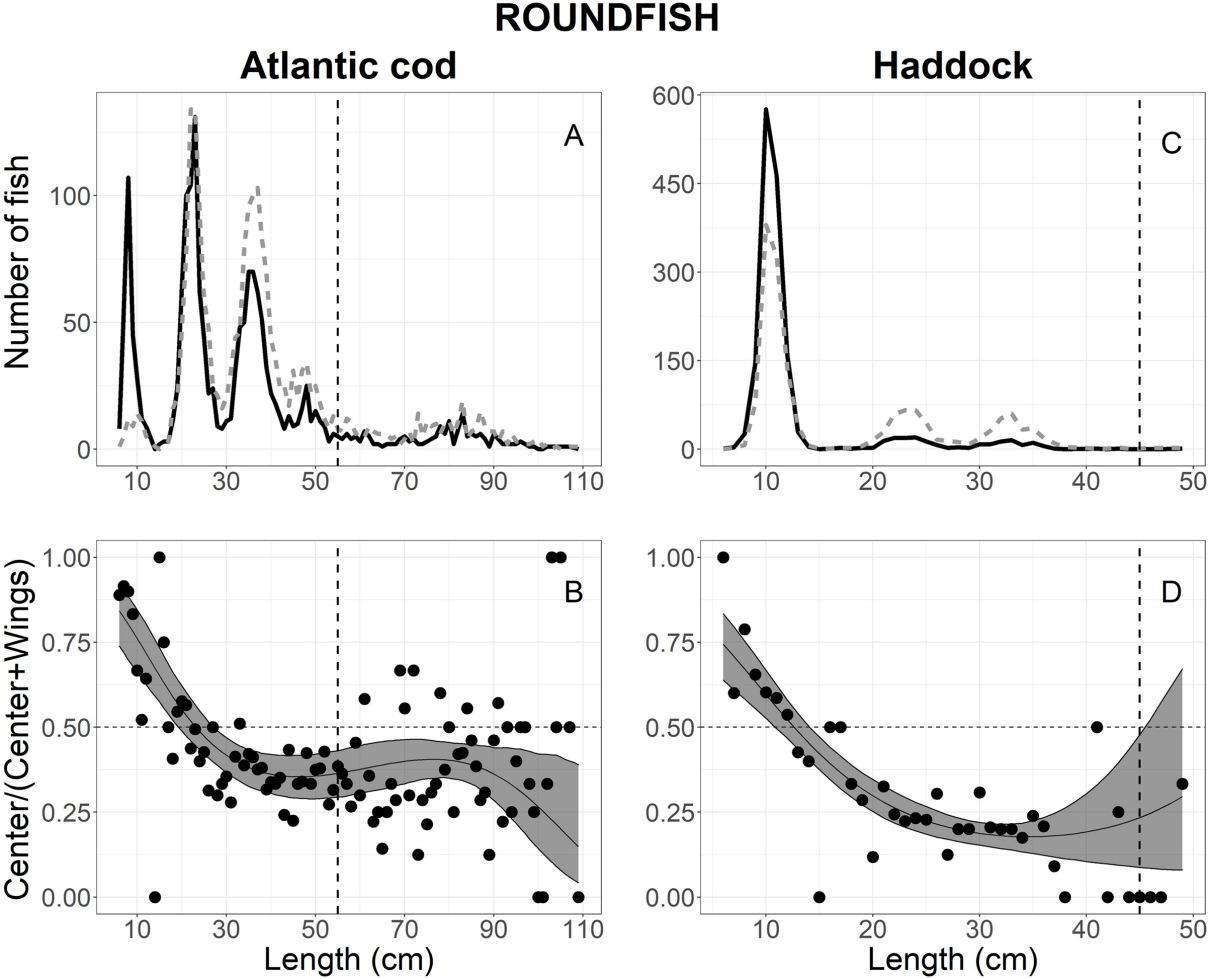
Length frequency and catch-at-length curves of roundfish. The length-frequency curves of (A) Atlantic cod and (C) haddock. The black line and grey dashed line represents the length frequencies of individuals in the center and both wing bags, respectively. The proportion curves at each length class for (B) Atlantic cod and (D) haddock. The black line represents the mean curves, and the grey shaded areas are the 95% confidence bands determined by bootstrap simulation. The vertical dashed line in each panel represents each fishery’s minimum reference length size. A value of 0.5 indicates an even split between collecting bags for the specific length.

A total of 3,796 Atlantic cod escaped into the collecting bags, 3,794 measured for analysis; 1,689 in the center, and 2,105 in the wings (Table 1). Figure 4A shows the size structure observed for Atlantic cod, where lengths ranged between 6 and 109 cm; most individuals were less than 55 cm (Fig. 4A). By comparing AICc values in Table 2, the best model was the logit-cubic and the random effect *Tow* on the intercept and quadratic slope (Table 3). The results showed that more Atlantic cod escaped in the center at lengths <20 cm than at the wings. Additionally, a large proportion (approximately 90%) of the smallest length classes escaped in the center. However, Atlantic cod >27 cm escaped at the wings significantly more often than the center with the highest catch proportion of 85% at the 110 cm length class (Fig. 4B).

**Table 2:** AICc values were estimated for several models.

Model	Independent variable and random effect	Atlantic cod	Haddock	European plaice	American plaice	Dab	Monkfish
Logit-constant	1	2,580.3	1,357.9	1,127.0	443.8	337.0	**245.7**
Logit-constant	1 + (1|*Tow*)	2,419.9	1,143.9	**1,093.4** *	**434.5** *	337.5	NA
Logit-linear *1*	*MLL* + (1|*Tow*)	2,381.2	943.4	**1,092.9**	**435.2**	339.5	NA
Logit-linear *2*	*MLL +* (*MLL|Tow*)	2,337.8	960.2	1,096.8	**435.6**	NA	NA
Logit-quadratic *1*	*MLL.orth.1 + MLL.orth.2 +* (*1|Tow*)	2,299.0	924.9	**1,093.9**	436.8	**334.5**	NA
Logit-quadratic *2*	*MLL.orth.1 + MLL.orth.2 +* (1 + *MLL.orth.1|Tow*) *+* (1+*MLL.orth.2|Tow*)	2,246.5	**910.0**	1,126.3	438.2	NA	NA
Logit-cubic *2*	*MLL.orth.1 + MLL.orth.2 + MLL.orth.3 + (*1 *+ MLL.orth.1|Tow) + (*1 *+ MLL.orth.2|Tow) +*	**2,221.7**	NA	1,105.5	442.8	NA	NA

**Notes:**

The bold number is the lowest AICc specifies the selected model.

* The AICc chosen as the simplest model between AICc values are similar or within 2 AICc.

NA is not applicable.

**Table 3:** GLMM parameters for catch-at-length comparison.

Species	Model	Parameter	Estimate	SE	*z*-value	*p*-value
Atlantic cod	Logit-quadratic *2*	${\beta_0}$	−0.325	0.098	−3.303	<0.001
		${\beta_1}$	5.727	1.647	−3.476	<0.001
		$\beta_2$	11.947	3.328	3.590	<0.001
Haddock	Logit-quadratic *2*	${\beta_0}$	−0.842	0.097	−8.672	<0.001
		${\beta_1}$	−13.521	1.607	−8.413	<0.001
		$\beta_2$	6.806	2.512	2.709	0.011
European plaice	Logit-constant	${\beta_0}$	−0.522	0.107	−4.887	<0.001
Dab	Logit-quadratic *1*	${\beta_0}$	0.156	0.152	1.026	0.304
		${\beta_1}$	−0.245	1.409	−0.174	0.861
		$\beta_2$	−3.563	1.358	−2.624	0.009
American plaice	Logit-constant	${\beta_0}$	−0.241	0.157	−1.540	0.123
Monkfish	Logit-quadratic *2*	${\beta_0}$	−1.294	0.191	−6.783	<0.001
		${\beta_1}$	−0.371	2.258	−0.164	0.870
		$\beta_2$	−2.774	2.860	−0.970	0.332

**Note:**

SE is the standard error of the estimate; estimate is the value of intercept or slope.

### Haddock

A total of 3,177 haddock escaped into the collecting bags, 3,175 measured for analysis; 1,585 in the center and 1,590 in the wings (Table 1). Fish length ranged between 6 and 50 cm; but most individual escapees were less than 15 cm (Fig. 4C). The escape-at-length curve of haddock was best described by the logit-quadratic and the random effect *Tow* on intercept and quadratic slope (Table 2), and the model’s parameters were shown in Table 3. The results show that haddock <11 cm escaped more often in the center than the wings, with the highest escape proportion of 75% at the smallest length class observed (Fig. 4D). By contrast, fish greater than 14 cm escaped significantly more in the wings than the center, and escapees increased in the wings with increasing length at 35 cm and up (Fig. 4D). For escapees >45 cm, large CIs were observed, attributing to the few individuals that escaped in the collecting bags and no significant difference was observed (Fig. 4D).

### European plaice

**Figure 5:**
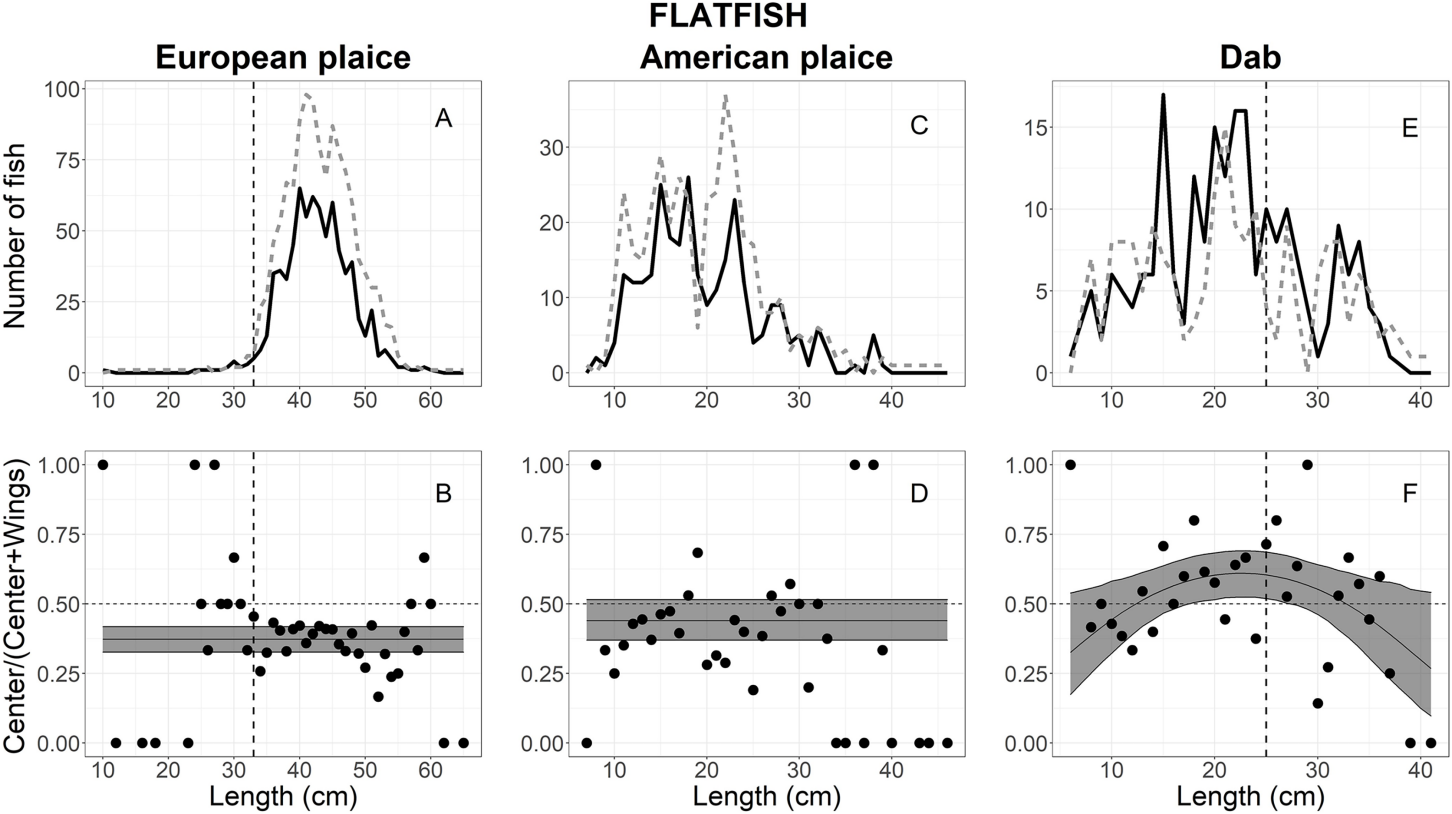
Length frequency and catch-at-length curves of flatfish. (A) European plaice, (C) American plaice, and (E) dab length-frequency curves. The black line and grey dashed line represent individuals length frequencies in the center and both wing bags, respectively. The proportion curves at each length class for (B) European plaice, (D) American plaice, and (F) dab. The black line represents the mean curves, and the grey shaded areas are the 95% confidence bands determined by bootstrap simulation. The vertical dashed line in each panel represents the minimum length size of each fishery. A value of 0.5 indicates an even split between collecting bags for the specific length.

A total of 2,025 European plaice escaped into the collecting bags, 1,958 measured for analysis; 739 in the center *vs* 1,219 in the wings (Table 1). The size structure observed for European plaice is shown in Fig. 5A, where lengths ranged between 10 and 65 cm; most individuals were between 30 and 55 cm (Fig. 5A). By comparing AICc values in Table 2, three models: logit-constant, logit-linear1, and logit-quadratic1, were within 2 AICc values, and the logit-constant model was then chosen since it was the simplest model (Table 3). The escape-at-length curve showed that significantly more (62.5%) European plaice escaped in the wings than in the center over all length classes (Fig. 5B).

### American plaice

Out of the 1,158 American plaice that escaped into the collecting bags, 685 were measured for analysis, including 279 in the center *vs* 406 in the wings (Table 1). The size structure observed for American plaice ranged between 7 and 46 cm; most individuals were between 10 and 30 cm (Fig. 5C). The AICc values in Table 2 showed that the logit-constant, logit-linear1, and logit-linear2 are good models to describe the experimental data, and the logit-constant model was then chosen since it was the simplest model (Table 3). The model indicated that American plaice escaped into the wings more often (55%) when compared to the center (45%), but these differences were not statistically significant as the CIs included 0.5 (Fig. 5D).

### Dab

For dab, a total of 405 individuals escaped into the collecting bags, 220 in the center and 185 in the wings (Table 1). The fish lengths ranged from 6 to 41 cm, with 10–35 cm having a high frequency (Fig. 5E). Based on AICc values (Table 2), the logit-quadratic with the random effect *Tow* on the intercept was the best model (Table 3). The escape-at-length curve was inflated at lengths between 18 and 33 cm by a few tows with large numbers at those sizes in the center compared with the wings (Fig. 5F). However, a significant difference was only found in the lengths between 18 and 27 cm, where the center had a little over half (54% at 23 cm) of the escaping dab (Fig. 5F). For the lengths <18 and >27 cm, the wing bags caught more fish than the center; but these differences were not statistically significant (Fig. 5F).

### Monkfish

**Figure 6:**
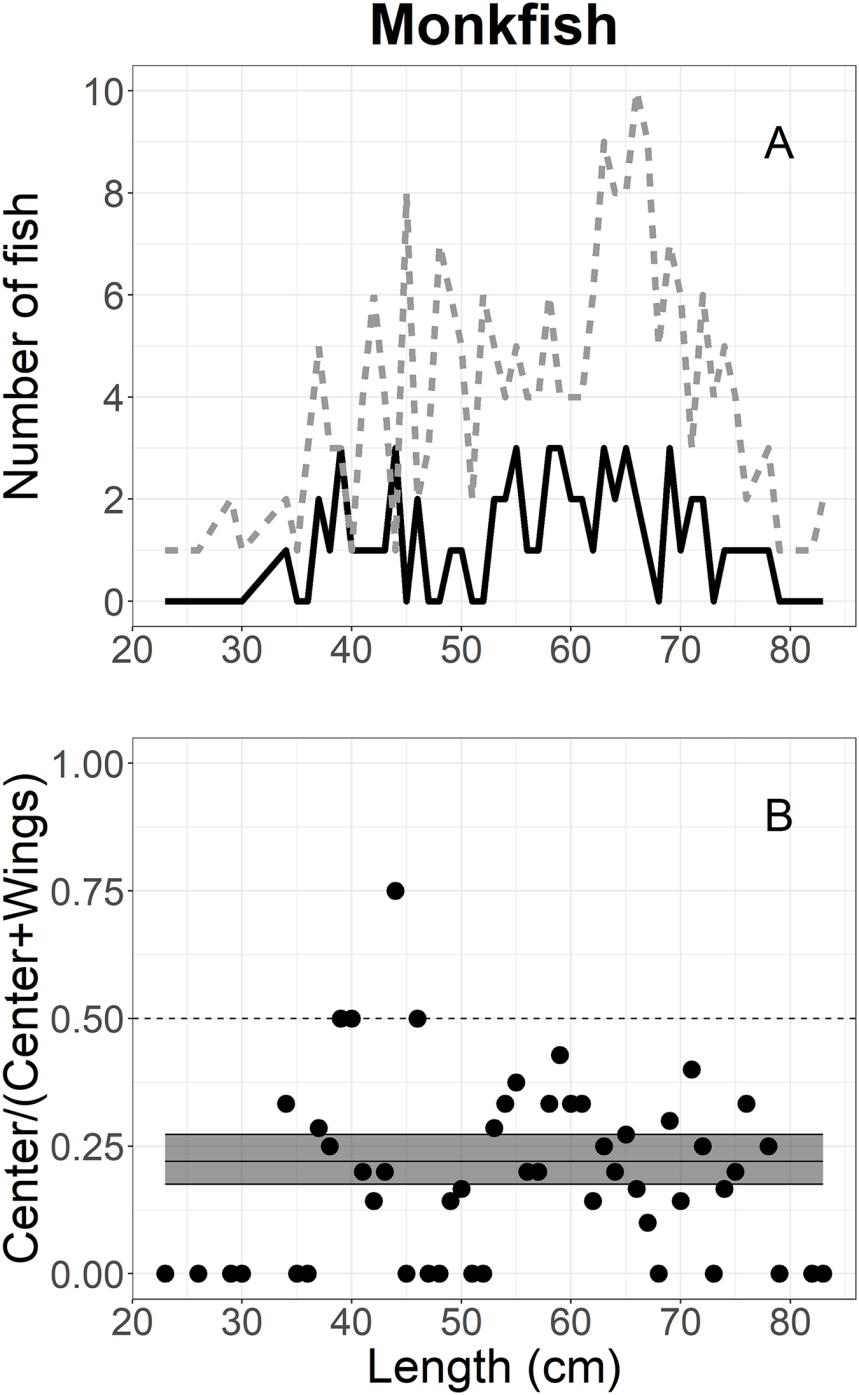
Length frequency and catch-at-length curves of monkfish. (A) The length-frequency curves of monkfish measured in collecting bags. The black line and grey dashed line represent individuals length frequencies in the center and both wing bags, respectively. (B) The proportions of escape at each length class estimated for monkfish. The black line represents the mean curves, and the grey shaded areas are the 95% confidence bands determined by bootstrap simulation. A value of 0.5 indicates an even split between collecting bags for the specific length.

The escape-at-length of monkfish was analyzed using 277 individuals escaping into the collecting bags during the experiment; 61 in the center and 216 in the wing (Table 1). Figure 6A shows the size structure of monkfish, where lengths ranged between 23 and 83 cm; most individuals were between 35 and 75 cm. Mixed models could not be used for this analysis due to singularity and convergence issues, likely due to the number of monkfish observed. Thus, generalized linear models were used as previously described with the random effect dropped and parametric CIs. The logit-constant model had the lowest AICc value (Table 2) and the model’s parameters were shown in Table 3. The proportion curve of monkfish was under 25% over all of the length classes, meaning that monkfish escaped more often in the wings (approximately 80% on average) than the center (approximately 20% on average), and these differences were statistically significant (Fig. 6B).

## Discussion

In this study, we quantified the length-dependent escape of fish at particular areas of the trawl mouth in terms of their response to herding effects, and swimming capacity. The analysis and results presented in this study were based on escape-at-length comparisons using the collecting bags method. Although the experimental design quantifies escapees under the fishing line, attaching the collecting bags potentially affects the fish’s behaviour during the herding process. Collecting bags mounted behind footgear might influence the gear geometry relative to standard commercial rigging. This could have affected fish behaviour or response to the trawl components, particularly the footgear. However, a previously published observation with a similar design revealed no abnormality in the door spread and headline height when attaching the collecting bags to the trawl (Ingólfsson & Jørgensen, 2006). In addition, Krag et al. (2010) tested a similar type of collecting bag system in a flume tank prior to their application in field research. Thus, the experimental trawl was assumed to be similar to the commercial trawl in trawl geometry, and the effects of collecting bags were regarded as negligible.

The escape-at-length analysis revealed a similar length-dependent escape under the trawl for Atlantic cod and haddock at different locations. The length-dependent escape by which smaller-sized individuals escaped more in the center for both Atlantic cod and haddock, <20 and 11 cm, respectively, whereas larger-sized fish (Atlantic cod >27 cm and haddock >14–46 cm) escaped more at the wings. If these escape-at-length curves were interpreted solely as a function of fish behaviour and/or swimming capacity at the trawl mouth, our study suggests that larger-sized fish likely were seeking to escape under the trawl at the wings, *vs* small fish being herded to the center and likely overran.

Main & Sangster (1981) initially observed that Atlantic cod remain close to the seabed in response to the approaching trawl components. However, recent observations have suggested that Atlantic cod probably swam or rose over the footgear as they passed through the net rather than remain close to the seabed (Pol & Eayrs, 2021; Thomsen, 1993). Combined with swimming capacity, these observations could explain the difference in escape behaviour of Atlantic cod in the trawl mouth based on sizes found between locations (center or wings). When aggregating in the center section, large Atlantic cod with greater swimming ability may rise above the fishing line, whereas small fish try to make escape attempts through spaces between rockhoppers of the footgear (Ingólfsson & Jørgensen, 2006; Pol & Eayrs, 2021; Ryer, 2008). An alternative explanation for the differing escape behaviour could be that if Atlantic cod react to the advancing trawl components late, they show erratic swimming, particularly near the trawl’s wings. This induces fish to be either run over by the footgear of the trawl or suddenly dart away by using a kick and glide gait (Brinkhof et al., 2017; Kim & Wardle, 2003). This response may lead to large fish escaping underneath the fishing line at the wing sections.

These results provided additional insights into Atlantic cod behaviour at the trawl mouth compared with prior studies. The escape behaviour of Atlantic cod related to the herding effect of the footgear was quantified by Walsh (1992), Ingólfsson & Jørgensen (2006), and in a more recent study by Krag et al. (2010). These studies used the mean catch data collected by the collecting bag method to compare the escape behaviour of Atlantic cod between center and wing areas of the footgear. The authors suggested that Atlantic cod more often escape the trawl from the center area rather than the wing areas as they aggregated in the front of the center part of the footgear in response to the herding effect (Wardle, 1993). In this study, we further found the escape rates at several particular lengths of Atlantic cod and compared those between escape locations, which could not be verified in previous studies.

Compared to Atlantic cod, the behavioural tendency of haddock at the trawl mouth has been observed to rise at heights above the fishing line as they entered the trawl net, confirming previous studies (Godø & Walsh, 1992; Main & Sangster, 1981). At least 74% of haddock escapees were less than 15 cm in length. This might indicate that the heights at which individuals rise at the trawl mouth depended on the individual’s swimming capacity, which differs according to the fish sizes. When herding at the center of the trawl mouth, small haddock (<11 cm) with poor swimming capacity may be seeking the spaces under the fishing line for escape relative to large individuals (>14 cm), or were simply run over by the trawl due to fatigue. This could be a plausible explanation that more small individuals were escaping from the center than the wings. In addition, a relatively high proportion of haddock >14 cm escaped through the wing areas, indicating that these haddock may illustrate an erratic response when they react to the approaching trawl components. Like Atlantic cod, the erratic response of haddock is represented by sudden darting away, kicking and gliding, or running over the footgear, leading to an increase in the number of large individuals escaping at the wings compared to the center.

The behavioural difference between Atlantic cod and haddock has played a vital role in developing separator trawls to separate Atlantic cod from the catch of haddock (Brinkhof et al., 2017; Krag et al., 2010; Larsen et al., 2018). Increasing spaces between the fishing line and the seabed can increase the escape of Atlantic cod under the trawl, therefore reducing bycatch of Atlantic cod from haddock-directed fisheries (Krag et al., 2010). Our results provided additional insights in which Atlantic cod >27 cm more often escaped at the wings. These results could potentially be used to develop new footgear to avoid capture of large Atlantic cod and small haddock.

The escape patterns of flatfish and monkfish between locations varied among species, and was less related to fish length. European plaice and monkfish were likely seeking to escape at the wings rather than the center, and escape rates were uniformed over length, indicating that the behaviour of European plaice was not related to fish length. Similarly, the slightly higher number of American plaice were likely attempting to escape at the wings rather than the center over the length, even though the difference was not statistically significant. In contrast, the escape of dab under the trawl was related to fish length, where a considerable proportion of fish >27 cm escaped more often in the center. These differences in escape patterns are probably due to behavioural differences, including escape behaviour and fish response to the herding effect of the trawl.

Most European plaice that escaped underneath the trawl was greater than 30 cm, including many commercial sizes (33 cm Minimum Conservation Reference Size). Their escape was observed more often at the outer footgear areas. This implied that the herding effect was not efficient to herd European plaice into the center of the footgear as flatfish herding behaviour was described by earlier studies (Bublitz, 1996; Main & Sangster, 1981). Bublitz (1996) observed that most flatfish left the substrate when oncoming nets approached and were generally herded into the center of the trawl mouth at different heights. The potential explanation for these differences may be the combination of response behaviour and swimming characteristics driven by fish densities (Godø, Walsh & Engås, 1999). At low densities, when the footgear is reached, flatfish, particularly medium and large individuals, swim ahead for a short distance in a zigzag pattern or swim laterally across the trawl mouth and then escape through the gaps under the wing sections of the footgear (Godø, Walsh & Engås, 1999; Winger et al., 2004; Bayse, Pol & He, 2016). Of note, the similar escape pattern observed for fish <30 cm should be considered with caution as the number of these fish were observed at a relatively low amount. Small European plaice with poorer swimming capacity become fatigued quickly during herding and are positioned still on the seabed when footgear passes above them (Ryer, 2008). This leads to a plausible consideration that the herding behaviour of small individuals did not differ between particular areas of the footgear.

Observations have shown that most American plaice escaping under footgear were less than 30 cm, and their escape was not significantly different between particular regions of the footgear (Winger, He & Walsh, 1999; Winger et al., 2004). These observations were consistent with the escape behaviour of American plaice found in the current study. The length-frequency analysis indicated that most American plaice escaped into collecting bags were smaller than 30 cm (Fig. 5C). The escape-at-length analysis could not reveal differences in escape behaviour of American plaice between locations along the footgear over observed lengths. These escape behaviours are likely explained based on the swimming capacity of fish, where small individuals with poor swimming capacity might prefer to bury in the substrate to escape rather than herd toward the center of the footgear (Winger, He & Walsh, 1999; Winger et al., 2004).

In contrast, the escape behaviour of dab was described by a slight bell-shaped escape-at-length comparison curve, where medium individuals (between 18 and 25 cm) escaped more often into the center of the footgear. More fish >12 cm escaped at the center than the wings, but a significant difference was found only between 18 and 25 cm (based on the lower CIs; Fig. 5F). This implied that the large individuals with greater swimming capacity likely exhibited a chain of behaviours involving swimming away and settling and were likely herded more often towards the center of the footgear than the wings. This finding is similar to Bayse, Pol & He (2016), who observed flatfish reactions to the footgear at the center area. The authors considered that likely large individuals may be overtaken by the trawl and probably escaped under the footgear. This escape behaviour was similar to the typical flatfish behaviour observed by Bublitz (1996) and reviewed by Ryer (2008).

Monkfish in the current study were observed to escape more often in the wings than the center over all length classes, confirming that monkfish were not herded into the center of footgear. This would suggest that there may be a camouflage behaviour of monkfish in relation to the gear during the herding process. It is similar to a flatfish response to the herding effect of the trawl described by Gibson (2005) and Winger, Eayrs & Glass (2010). Monkfish likely have poor swimming ability and may swim less than the speed of advancing sweeps, remain in their positions on the seabed briefly, or bury in the substrate. This strategy may allow monkfish to keep their positions close to the wing sections rather than herding into the center of the footgear.

Our findings provide more details on the size-specific behaviour of fish at the trawl mouth compared with previous studies. Most recorded video projects have directly interpreted fish behaviour mainly in the narrow area at the center of the footgear (Kim & Wardle, 2003; Winger et al., 2004; Underwood et al., 2015; Bayse, Pol & He, 2016). Here, our study quantified the extent to which fish react to the footgear between the center and outer areas (wings) with a length-based approach. This analysis provides higher precision to fish behaviour in the trawl when compared to Walsh (1992) and Ingólfsson & Jørgensen (2006), who quantified differences in escape behaviour between different regions of the footgear using an analysis that combined the catch of all areas under the trawl. Based on the results obtained, the fish behaviour at the center of footgear in this study was generally consistent with those quantitatively described by underwater video analysis. This supports that the escape-at-length comparison method used in this study can be employed to infer fish behaviour in relation to the herding effect of the trawl. Future analysis of fish behaviour at the wing areas could include different combinations of this method with underwater cameras to improve understanding of the complex behaviour sequence of fish that lead to fish escaping under the trawl at the wing areas. Additionally, this method would be useful for studies where cameras are not an option due to low visibility or extremely rough sea beds.

## Conclusions

This study used the escape-at-length comparison method to infer fish behaviour at different escape locations under the footgear of a trawl. Overall, fish behaviour was characterized by escape behaviours, herding response, and swimming capacity, which varied among species and differed according to fish size. The behaviour of roundfish such as Atlantic cod and haddock quantified in the current study is length-dependent and agrees with previous observations. Flatfish reaction at the trawl mouth showed more variation with species-specific results than roundfish. While the escape behaviour of dab found in this study was similar to common flatfish’s behaviour observed by previous underwater observations (Bayse, Pol & He, 2016; Bublitz, 1996; Ryer, 2008), some flatfish such as European plaice and American plaice exhibited contrary results. These unexpected results need further observations to improve the understanding of how fish respond to the herding effect of the trawl.

Developing the footgear of a trawl to improve the trawl’s selectivity based on fish escape under the fishing line can be one of the important options available for fisheries management (Engås & Godø, 1989; Walsh, 1992; Ramakrishnan, 2018). The rockhopper footgear is both effective at catching fish close to the seabed while also allowing the escape of small gadoid fish (Engås, Jacobsen & Soldal, 1988; Ingólfsson & Jørgensen, 2006). Small fish have been observed to actively search the escape opening along the footgear under the fishing line between rockhopper discs (Engås, Jacobsen & Soldal, 1988). Fish behaviour inferred in this study is in line with those observations while providing new findings of small gadoid fish escaping more often in the center area. This suggests that modifications to the footgear regarding increasing spaces between the fishing line and the bottom at particular locations might increase the escape of undersized fish and flatfish, which generally enter the trawl at heights less than 1 m. Alternatively, increasing escaping spaces may reduce fish encounters with the footgear, which is considered to be a cause of mortality of fish when escaping under the trawl (Ingólfsson & Jørgensen, 2006).

## Supplemental Information

10.7717/peerj.14746/supp-1 Supplemental Information 1 Raw data, separated data of selected species, and R codes that refer to the separated data. Raw data includes the fish length data measured for all species captured by main codend and collecting bags and the trawl operation data recored the trawl operation parameters and fishing conditions. The fish length data of each selected species was separated through the R codes included.

10.7717/peerj.14746/supp-2 Supplemental Information 2 A schematical drawing of the collecting bag experiment. (A) and (B) are the side and top view of collecting bags. This schematical drawing is modified from Ingólfsson & Jorgensen (2006).

## Additional Information and Declarations

### Data Availability

The following information was supplied regarding data availability: The raw data measurements are available in the Supplemental Files.
