# Supplementary figures and images for: Inferring fish behaviour at the trawl mouth from escape location
Source: PeerJ. 2023 Jan 25;11:e14746. doi: 10.7717/peerj.14746 (PMC9884032; doi:10.7717/peerj.14746)

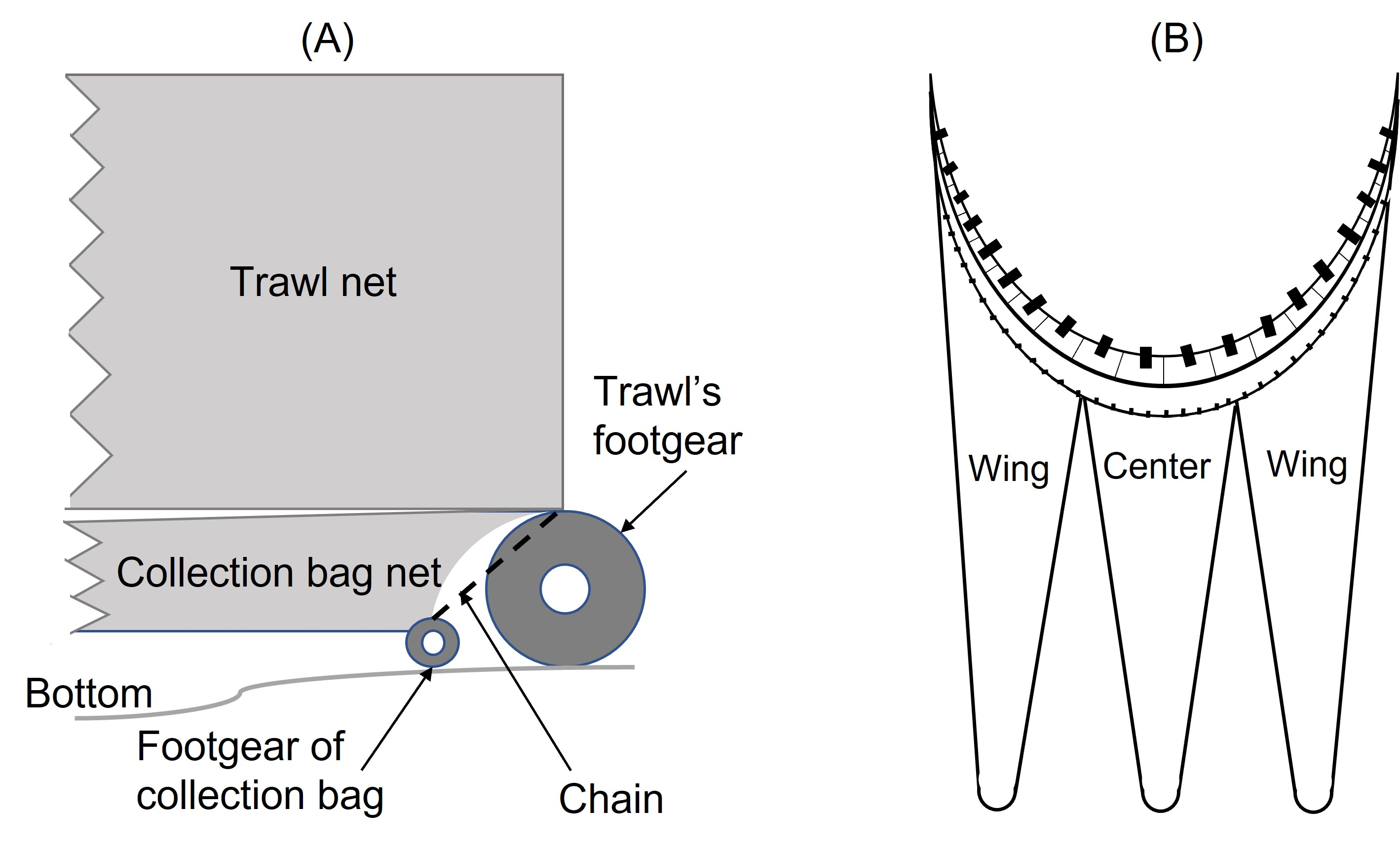

Supplement: Supplemental Information 2 — (A) and (B) are the side and top view of collecting bags. This schematical drawing is modified from Ingólfsson & Jorgensen (2006). [file peerj-11-14746-s002.png]
